# International Classification of Functioning, Disability, and Health-based rehabilitation program promotes activity and participation of post-stroke patients

**DOI:** 10.3389/fneur.2023.1235500

**Published:** 2023-11-03

**Authors:** Mabel Ngai-Kiu Wong, Mike Kwun-Ting Cheung, Yuk-Mun Ng, Huan-Ling Yuan, Bess Yin-Hung Lam, Siu Ngor Fu, Chetwyn Che Hin Chan

**Affiliations:** ^1^Department of Rehabilitation Sciences, The Hong Kong Polytechnic University, Kowloon, Hong Kong SAR, China; ^2^Department of Psychology, The Education University of Hong Kong, New Territories, Hong Kong SAR, China; ^3^Centre on Research and Advocacy, The Hong Kong Society of Rehabilitation, Hong Kong, Hong Kong SAR, China; ^4^Rehabilitation Division, The Hong Kong Society for Rehabilitation, Hong Kong, Hong Kong SAR, China; ^5^Department of Counselling and Psychology, Hong Kong Shue Yan University, Hong Kong, Hong Kong SAR, China

**Keywords:** goal-setting process, multidisciplinary approach, community reintegration, resuming life roles, stroke rehabilitation

## Abstract

**Background:**

The International Classification of Functioning, Disability, and Health (ICF) model has been applied in post-stroke rehabilitation, yet limited studies explored its clinical application on enhancing patients’ Activity and Participation (ICF-A&P) level.

**Purpose:**

This study gathered evidence of the effects of an ICF-based post-stroke rehabilitation program (ICF-PSRP) in enhancing community reintegration in terms of ICF-A&P of post-stroke patients.

**Methods:**

Fifty-two post-stroke patients completed an 8 to 12 weeks multidisciplinary ICF-PSRP after setting personal treatment goals in an outpatient community rehabilitation center. Intake and pre-discharge assessments were administered for primary outcomes of Body function (ICF-BF; e.g., muscle strength) and ICF-A&P (e.g., mobility), and secondary outcomes of perceived improvements in ability (e.g., goal attainment and quality of life).

**Results:**

There were significantly higher levels in the ICF-BF and ICF-A&P domains, except cognitive function under the ICF-BF. Improvements in the primary outcomes predicted corresponding secondary outcomes. Firstly, expressive and receptive functions (ICP-BF) were mediated by the everyday language (ICF-A&P) which predicted patients’ satisfaction with the language-related quality of life. Secondly, upper extremity function (ICP-BF) was mediated by the lower extremity mobility (ICF-A&P) predicting work and productivity-related quality of life. Content analyses showed that combined ICF-BF and ICF-A&P contents throughout the ICF-PSRP contributed to the positive treatment effects.

**Conclusion:**

The ICF-PSRP was effective in promoting body function, and activity and participation levels of post-stroke patients. Positive treatment effects are characterized by goal-setting process, cross-domain content design, and community-setting delivery.

**Clinical trial registration**: https://clinicaltrials.gov/study/NCT05941078?id=NCT05941078&rank=1, identifier NCT05941078.

## 1. Introduction

Stroke is a neurological disease and one of the leading causes of death and disability globally (1). Post-stroke patients often encounter disabilities, such as limitations in their activities of daily living (ADL) and cognitive impairment (2). Studies have described multidisciplinary post-stroke rehabilitation programs (3) and their positive effects on post-stroke patients’ community integration and quality of life (4). Other post-stroke rehabilitation programs have targeted the promotion of patients’ independence in ADL (5) and instrumental activities of daily living (IADL) (6). These studies have emphasized an eclectic approach to maximizing the regaining of function and independence after stroke. Adopting a comprehensive framework, such as the International Classification of Functioning, Disability, and Health (ICF) (7), may offer a systematic approach to the provision of post-stroke rehabilitation. The ICF model emphasizes activity and participation (ICF-A&P) as the core concepts of rehabilitation. Activity refers to functioning at the individual level (e.g., ADL), while participation refers to functioning in all areas of life (e.g., IADL) (8). The conventional body function (physiological; ICF-BF) and body structure (human anatomical parts) components are the building blocks that support the ICF-A&P. These three components are affected by two contextual factors: Personal (PF) and Environmental factors (EF). The ICF model has been widely applied in the design of assessments, patient profiles, and treatment approaches in different rehabilitation disciplines (9, 10).

Applications of the ICF model to design rehabilitation programs for post-stroke patients are scarce. A review of the existing literature only identified three ICF-based studies. A case study by Abarghuei et al. (11) reported the effect of a 1 month occupational therapy program for a middle-aged man with chronic stroke. The ICF Core Set (ICF-CS) for stroke was deployed in the assessments, personalized goals were set, and the treatment contents were administered to enhance independent community living. The patient’s outcomes were improvements in muscle power and muscle tone, the ability to walk up and down stairs, and outdoor mobility without assistance. A second case study by Begum and Haque (12) involved an ICF-based physiotherapy program for a female post-stroke patient. The ICF-CS was used to identify the patient’s problems and set goals. The results showed improvements in the balance and shoulder mobilization components of the ICF-BF and the walking ability component of the ICF-A&P after the 3 months treatment program. For the third study, Mehraban et al. (13) designed an ICF-based 2 months occupational therapy program with an approach comparable to that of Abarguhei et al. (11). When compared with patients in the usual practice control group, those in the ICF-based program showed improvements in motor function and satisfaction with their level of productivity in paid/unpaid work and household management and their leisure activities. All three studies described above were operationalized by a single rehabilitation discipline, which may limit the scope covered by the wide spectrum of contents covered in an ICF-based rehabilitation program. More importantly, the limited number of studies indicates the need to further investigate the clinical applications of the ICF model in post-stroke rehabilitation.

The aim of the current study was to design and gather evidence on the effects of the first ICF-and community-based multidisciplinary rehabilitation program for post-stroke patients in Hong Kong. We used a pre-and post-treatment design to evaluate the changes in the patients’ ICF-A&P and ICF-BF levels. Besides, we focused on the relationships between the ICF-A&P and ICF-BF embedded in the treatment contents, and the patients’ subjective satisfaction with the treatment outcomes. We hypothesized that post-stroke patients would show improvements in both ICF-A&P and ICF-BF measures at the end of the program. Moreover, we hypothesized significant relationships between the ICF-A&P and ICF-BF components, which would contribute to the patients’ subjective satisfaction with the treatment outcomes.

## 2. Materials and methods

### 2.1. Participants

Post-stroke patients were recruited from a community-based rehabilitation program operated by a non-governmental organization in Hong Kong. Patients were included if they (1) had a diagnosis of stroke with an onset no more than 24 months, (2) were medically stable, (3) were able to transfer or walk with no more than one item of assistance, and (4) were able to tolerate at least 2 h of active rehabilitation treatment. Thirty-three (63.5%) of the participants were male, and the ages of the participants ranged from 34 to 78 years (mean = 56.1 years, standard deviation [SD] = 10.6). The mean duration since the stroke was 7.8 months (SD = 5.7). All participants voluntarily participated in the study and provided informed consent. This study followed the Declaration of Helsinki and was approved by the Human Subjects Ethics Sub-committee of Hong Kong Polytechnic University (HSEARS 20210407006).

### 2.2. Setting

The ICF-based post-stroke rehabilitation program (ICF-PSRP) under the study was an outpatient service provided in a community rehabilitation center. The rehabilitation center is operated by a non-government organization. It was targeted to facilitate patients’ functional improvement, and community and social reintegration. Duration of the ICF-PSRP was eight or 12 weeks, comprising of 30 or 48 two-hour sessions depending on the patients’ needs and progress. The goal-setting process, intervention contents, pathways and flow, and assessments were based on the ICF-CS for Stroke as the framework. The ICF Assessment Sheet was adopted to document the patients’ conditions as well as the records of the goals set and progress made in the treatment sessions. Delivery of the ICF-PSRP was by a multi-disciplinary team composed of occupational therapists (OT), physiotherapists (PT), and speech therapists (ST) in a community rehabilitation centre.

**Figure 1:**
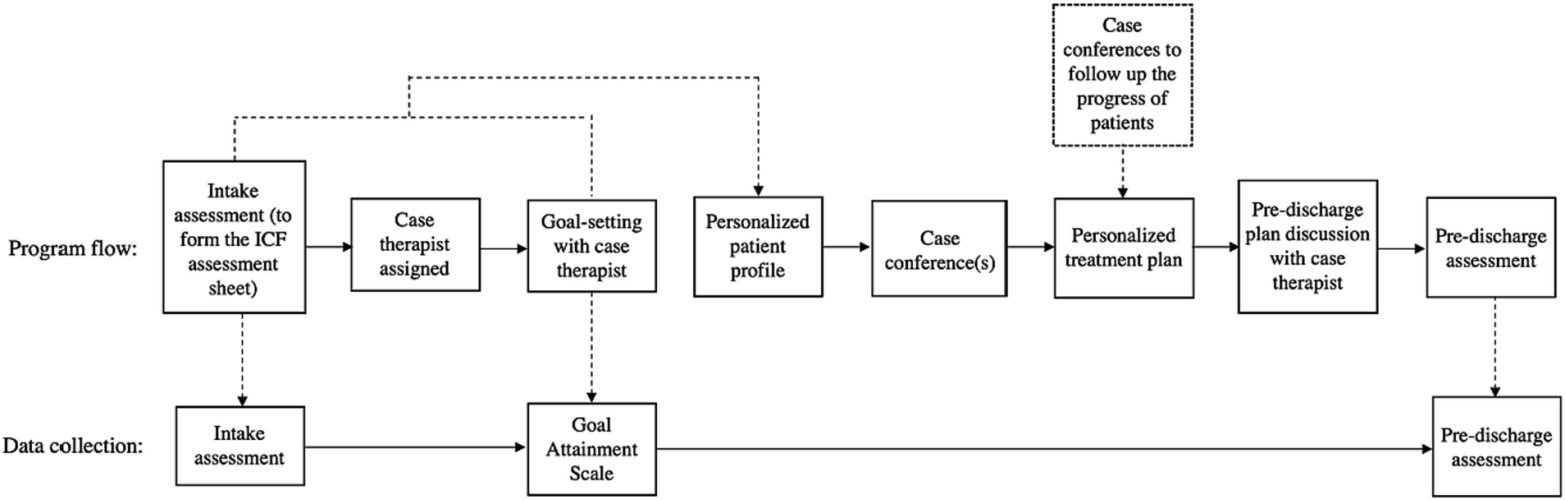
The flow diagram of the ICF-based post-stroke rehabilitation program.

After admission, the patients completed the intake assessment with therapists in different disciplines. Each patient was then allocated a case therapist based on the assessment results. A one-on-one intake interview with a case therapist assigned to the patient was to facilitate the patient to set personal treatment goals (Figure 1). The patients (and their caregivers, if any) discussed their treatment goal(s) with the case therapist. The goal setting interview began with the case therapist asking guiding questions on patients’ life roles, challenges with daily routines, and then short-and long-term goals. The case therapist explained the meanings of the ICF-A&P in layman’s terms. After goals were listed, the case therapist guided the patient to set their priority and integrate them into the daily treatment sessions. The goals were documented in the goal attainment scale. In the monthly case conference, the program team composed personalized treatment plans for patients based on the information gathered from the intake interviews. Treatment plan included prescriptions of specific intervention modules with set intensities and durations. Progresses made by patients in terms of assessment results (see below) and updates of treatment plans or discharge plans were also covered in the case conferences.

The treatment program contents were organized as ICF-A&P and ICF-BF intervention modules. Patients’ EF and PF concerns were covered in the goal-setting interview. The issues identified and of high priority would be tackled with interventions implemented during the treatment sessions, or by other professionals such as nurses (for chronic diseases apart from stroke) and social workers (for financial difficulty) at the center. Each of the OT, PT, and ST disciplines set the aims and developed the training contents, intensities, durations, and upgrading and completion criteria. There were eight modules in the ICF-A&P domain and another eight modules in the ICF-BF domain. The PT discipline offered modules such as strengthening exercises (ICF-BF contents) and gait training (ICF-A&P contents). The OT discipline offered modules such as strengthening exercises (ICF-BF contents) and self-care training (ICF-A&P contents). The ST discipline offered such as oro-motor (ICF-BF contents) and communication training (ICF-A&P contents). The community training module is to involve multi-disciplinary efforts on transportation and shopping training (ICF-A&P contents).

### 2.3. Materials

Clinical outcomes adopted in this study referred to the ICF-CS for Stroke contents. However, the ICF-PSRP did not use ICF Categorical Profile as main outcome measure because of potential novel-rater reliability biases reported in a previous study (14). Instead, standardized discipline-based instruments mapped to the ICF-CS for Stroke contents were deployed as the outcome measures (Table 1). The primary outcome measures were the Chinese version of the Modified Barthel Index (mBI-C), Chinese version of the Lawton Instrumental Activities of Daily Living (iADL-CV), Elderly Mobility Scale (EMS), Therapy Outcome Measure (TOM), Manual Muscle Testing – Lower Extremity (MMT-LE), Hong Kong version of the Functional Test for the Hemiplegic Upper Extremity (FTHUE-HK), Hong Kong version of the Oxford Cognitive Screen (HK-OCS). The Goal Attainment Scale (GAS) and subscales of the Stroke Specific Quality of Life Scale (SSQoL-C) were the secondary outcome measures. The outcomes of the ICF-PSRP were set basing on the components stipulated in the ICF Stroke Core Set. The research team intended to compare the results of the study with other non-ICF post-stroke rehabilitation studies. As a result, text constructs of common clinical instruments guided mapping with the Stroke ICF components. For example, items of the “writing ability” item of the Lawton Instrumental Activities of Daily Living Scale was mapped to the “d170 Writing” of the ICF Activity and Participation component, and the “washing dishes” was mapped to the “d640 Doing housework” (8).

**Table 1:** The mapping of standardized clinical instruments on the ICF Body Function (ICF-BF) and Activity and Participation (ICF-A&P) components administered by the occupational therapists (OT), physiotherapists (PT), and speech therapists (ST) in the ICF-PSRP.

ICF categories	Clinical instruments
Primary Outcome Measures – ICF Body Function	Hong Kong version of the Functional Test for the Hemiplegic Upper Extremity (FTHUE-HK; OT)
	Hong Kong version of the Oxford Cognitive Screen (HK-OCS; OT)
	Manual Muscle Testing – Lower Extremity (MMT-LE; PT)
	Therapy Outcome Measures (TOM) – Impairment scale (ST)
Primary Outcome Measures – ICF Activity and Participation	Chinese Version of the Lawton Instrumental Activities of Daily Living Scale (iADL-CV; OT)
	Chinese version of Modified Barthel Index (mBI-C; OT)
	Elderly Mobility Scale (EMS; PT)
	Therapy Outcome Measures (TOM) – Disability, Handicap, Well-being scales (ST)
Secondary outcome measures	Chinese version of the Stroke Specific Quality of Life Scale (SSQoL-C) – Family role, Language, Mobility, Self-care, Social role, Upper extremity function, Work and productivity subscales (CASE)
	Goal Attainment Scale (GAS; CASE-intake)

CASE refers to case therapists administering the instrument to the patients. CASE-intake refers to case therapists administering the instrument to the patients during the intake interview.

ICF-A&P (ADL) (15). The Chinese version of the Modified Barthel Index (mBI-C) measures the level of self-care management activities (16, 17). It has shown moderate to strong test–retest reliability in post-stroke patients (Kappa value >0.60).

ICF-A&P (IADL) (8). The Chinese version of the Lawton Instrumental Activities of Daily Living (iADL-CV) Scale measures the level of independent living (18, 19). Its inter-rater and test–retest reliability have demonstrated intra-class correlation coefficient (ICC) values greater than 0.90.

ICF-A&P (mobility) (20). The EMS measures the mobility level (21, 22) with satisfactory test–retest reliability (ICC > 0.87) (23).

ICF-A&P/ICF-BF [expressive and receptive languages (ERL)]. The Therapy Outcome Measure (TOM) assesses patients’ abilities and difficulties in terms of their impairment, activity, participation, and well-being in ERL abilities (24). The impairment scale refers to the ICF-BF, while the Activity, Participation, and Well-Being scales refer to the ICF-A&P (24). The instrument has shown high ICCs (>0.70) (24, 25).

ICF-BF (LE) (26). The Manual Muscle Testing – Lower Extremity (MMT-LE) scale measures muscle strength impairments (27). The MMT-LE has shown good reliability and validity (28).

ICF-BF (UE) (29). The Hong Kong version of the Functional Test for the Hemiplegic Upper Extremity (FTHUE-HK) measures recovery of the hemiplegic UE (30, 31). The test has shown high sensitivity and specificity, item-level correlation (*r* > 0.71), and internal consistency (*α* > 0.840) in post-stroke patients.

ICF-BF (cognition) (29). The Hong Kong version of the Oxford Cognitive Screen (HK-OCS) measures stroke-induced cognitive disabilities (32, 33). The test has been validated in post-stroke patients in Hong Kong, with strong concurrent validity (*r* > 0.50), fair test–retest reliability (*α* < 0.80) for most subtests, and acceptable internal consistency (*α* = 0.725).

Goal attainment. The GAS was used to enable patients to set achievable goals at the beginning of the program (34). The goals were set according to the patients’ functional gaps and life roles prior to stroke onset via interactions with therapists. The scale has been found to reflect changes in the extent of achieving set goals among post-stroke patients (35).

Quality of life. The Chinese version of the Stroke Specific Quality of Life Scale (SSQoL-C) measures post-stroke patients’ health-related quality of life with good internal consistency (*α* > 0.63) and acceptable convergent validity (Spearman’s rho >0.40) (36, 37). For subscales of the SSQoL-C, our program focused on A&P-based outcomes; thus, only those identified in that category were assessed.

### 2.4. Study design and data collection procedures

This study employed a quasi-experimental, within-subject design. Patient recruitment and study implementation were completed in April 2021 to July 2022. There were two waves of data collection: at intake and prior to discharge. The patients completed intake assessments within 3 weeks before the start of the program, depending on their availability. Therapists in the ICF-PSRP team administered the intake assessments (Table 1). The intake interview was conducted by the assigned case patient which covered demographic information and medical and rehabilitation service histories. The case therapist also guided the patient (and their caregivers, if any) to set treatment goals within the first to fourth sessions of the program. The assessment results and patients’ goals were reported in the team case conference in which treatment program plan was formulated. In the final four sessions of the program (i.e., the 27th to 30th or 45th to 48th sessions), the case therapist conducted assessments on the goal attainment and pre-discharge assessments using the same set of instruments used in the intake assessments.

### 2.5. Data analysis

Scoring of the clinical instruments followed the method stipulated in the test manuals except the MMT-LE of which scores were first transformed according to the method described by Bohannon (27) before computing the mean score. Missing data were replaced by the expectation-maximization method. One-way repeated-measure analysis of variance was used to test the significance of the differences in the scores of the primary and secondary outcomes between the two assessment occasions. To test the possible effects of patients’ gender on the outcomes, gender was entered as a covariate in the one-way repeated-measure analysis of covariance model tested for its significance on the assessment score changes. Bonferroni adjustments were applied to the 0.05 significance level to control for potential type I errors (38).

**Figure 2:**
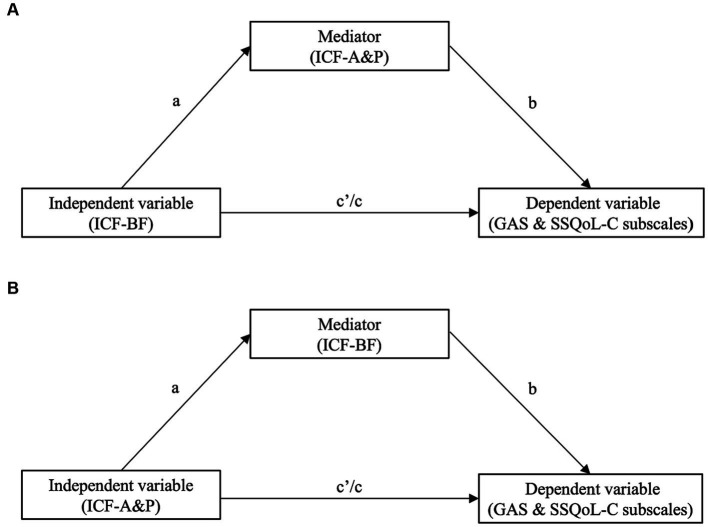
The conceptual model describing the relationships among the ICF-BF, ICF-A&P and secondary outcomes [i.e., Goal Attainment Scale (GAS) and subscales in the Chinese version of the Stroke Specific Quality of Life Scale (SSQoL-C)] of the ICF-based post-stroke rehabilitation program. The change in the independent variable contributes to the secondary outcomes as the dependent variable, which is controlled by the mediator. **(A)** ICF-BF as the independent variable and ICF-A&P as the mediator contributing to secondary outcomes. **(B)** ICF-A&P as the independent variable and ICF-BF as the mediator contributing to secondary outcomes. In both models, the a, b and c paths are indirect effects, and the c′ path is a direct effect.

Mediation analyses were conducted using the PROCESS tool (version 4.1) (38). Two models were tested. The first model was on the ICF-A&P (mediator) and ICF-BF (independent variable, IV) scores, while the second model was on the ICF-BF (mediator) and ICF-A&P (IV) scores. Both models included secondary outcomes as the dependent variable (DV; i.e., GAS and SSQoL-C subscale scores). Figure 2 presents the speculated relationships between the variables. All measures for the ICF-A&P and ICF-BF were change scores between the two assessment occasions (39). Covariates for the models were the age and gender of the patients. Significant models were combined and tested using structural equation modelling (SEM) using IBM SPSS Amos (version 28). The data-to-model fits were assessed based on the results of chi-square tests, and root mean square error approximations, the comparative fit, and the goodness-of-fit index were indicators used to assess the fit of the model.

A second-level analysis was then conducted to triangulate the SEM models with the treatment modules that the patients completed. Qualitative content analyses of the scheduling of the ICF-A&P and ICF-BF treatment modules would shed light into how the timing and sequential relationships between these two types of training modules contributed to the secondary treatment outcomes. The ICF-PSRP for each patient was divided into three phases: beginning (i.e., the 1st to 10th sessions of a 30-session program or the 1st to 16th sessions of a 48-session program), middle (i.e., the 11th to 20th or 17th to 32nd sessions), and late (i.e., the 21st to 30th or 33rd to 48th sessions). The timing/sequence of the training modules delivered to each patient were coded for each of the three phases. There were three delivery scenarios: (1) ICF-BF before ICF-A&P modules (i.e., BF➔A&P), (2) ICF-A&P before ICF-BF modules (i.e., A&P➔BF), and (3) concurrent ICF-BF and ICF-A&P modules (i.e., BF|A&P). The module sequences and the number of patients in each sequence were collated and compared and related to the results of the SEM models.

## 3. Results

### 3.1. Demographics

Seventy-four patients were invited and 58 of them agreed to participate in the study. Six patients were excluded due to a second stroke, program withdrawal and suspension, leaving 52 patients included in the data analysis. Of the 52 patients, 22 reported having an ischemic stroke, and 24 reported having a hemorrhagic stroke. One patient reported having an ischemic stroke followed by a hemorrhagic complication, and five patients did not indicate their type of stroke. Twenty-six patients (50%) had left hemiplegia, 24 patients (46.2%) had right hemiplegia, and two patients had diplegia (Table 2).

**Table 2:** Demographic characteristics of the post-stroke patient participants.

Variables	*N* (%)
Age range (in years)	34 to 78
Mean age (in years; SD)	56.1 (10.6)
Gender (SD)	
Male	33 (63.5)
Female	19 (36.5)
Types of stroke	
Ischemic stroke	22 (42.3)
Hemorrhagic stroke	24 (46.2)
Ischemic stroke with hemorrhagic complication	1 (1.9)
Not indicated	5 (9.6)
Months since stroke* (SD)	7.8 (5.7)
Side of hemiplegia	
Left	26 (50.0)
Right	24 (46.2)
Bilateral	2 (3.8)

SD, standard deviation. *Calculated between the date of the stroke to the date of completing the intake assessment.

### 3.2. Treatment effects on body function

Patients completing the ICF-PSRP showed significant improvements in scores for the FTHUE-HK (*p* < 0.001); HK-OCS Attention subtest (*p* = 0.017); MMT-LE (*p* < 0.001); and TOM impairment scales for receptive aphasia (*p* = 0.017), expressive aphasia (*p* < 0.001), and dysarthria (*p* < 0.001; Supplementary Table S1). No significant changes were observed in the other HK-OCS subtests (*p* > 0.05). After Bonferroni adjustments (*p* = 0.0125), significant changes were found in the scores for the FTHUE-HK, MMT-LE, and TOM impairment scales for expressive aphasia and dysarthria.

### 3.3. Treatment effects on activity and participation

After completing the ICF-PSRP, the patients showed significant improvements in scores for the EMS (*p* < 0.001); iADL-CV (*p* < 0.001); mBI-C (*p* < 0.001); TOM disability scales for receptive aphasia (*p* < 0.001), expressive aphasia (*p* < 0.001), and dysarthria (*p* < 0.001); TOM handicap scales for receptive aphasia (*p* < 0.001), expressive aphasia (*p* < 0.001), and dysarthria (*p* < 0.001); and TOM well-being scales for receptive aphasia (*p* = 0.005), expressive aphasia (*p* < 0.001), and dysarthria (*p* < 0.001; Supplementary Table S1). Similarly, after Bonferroni adjustments (*p* < 0.0125), statistically significant improvements persisted for all the above mentioned A&P-based clinical instruments showing pre-and post-treatment changes.

### 3.4. Treatment effects on secondary outcomes

Significant improvements were also observed for secondary outcomes in patients completing the ICF-PSRP. Scores for the GAS (*p* < 0.001) and the SSQoL-C subscales of Family Role (*p* = 0.047), Language (*p* < 0.001), Mobility (*p* = 0.004), Social Role (*p* = 0.001), and Work and Productivity (*p* = 0.037) were significantly improved after completing the ICF-PSRP (Supplementary Table S1). Changes in the scores for the SSQoL-C subscale of the Upper Extremity scale were marginally statistically significant (*p* = 0.055).

### 3.5. Gender as the covariate

The gender as the covariate was found not statistically significant on patients’ changes in all the outcome measures between the intake and pre-discharge assessments.

### 3.6. Mediation and SEM analyses

**Figure 3:**
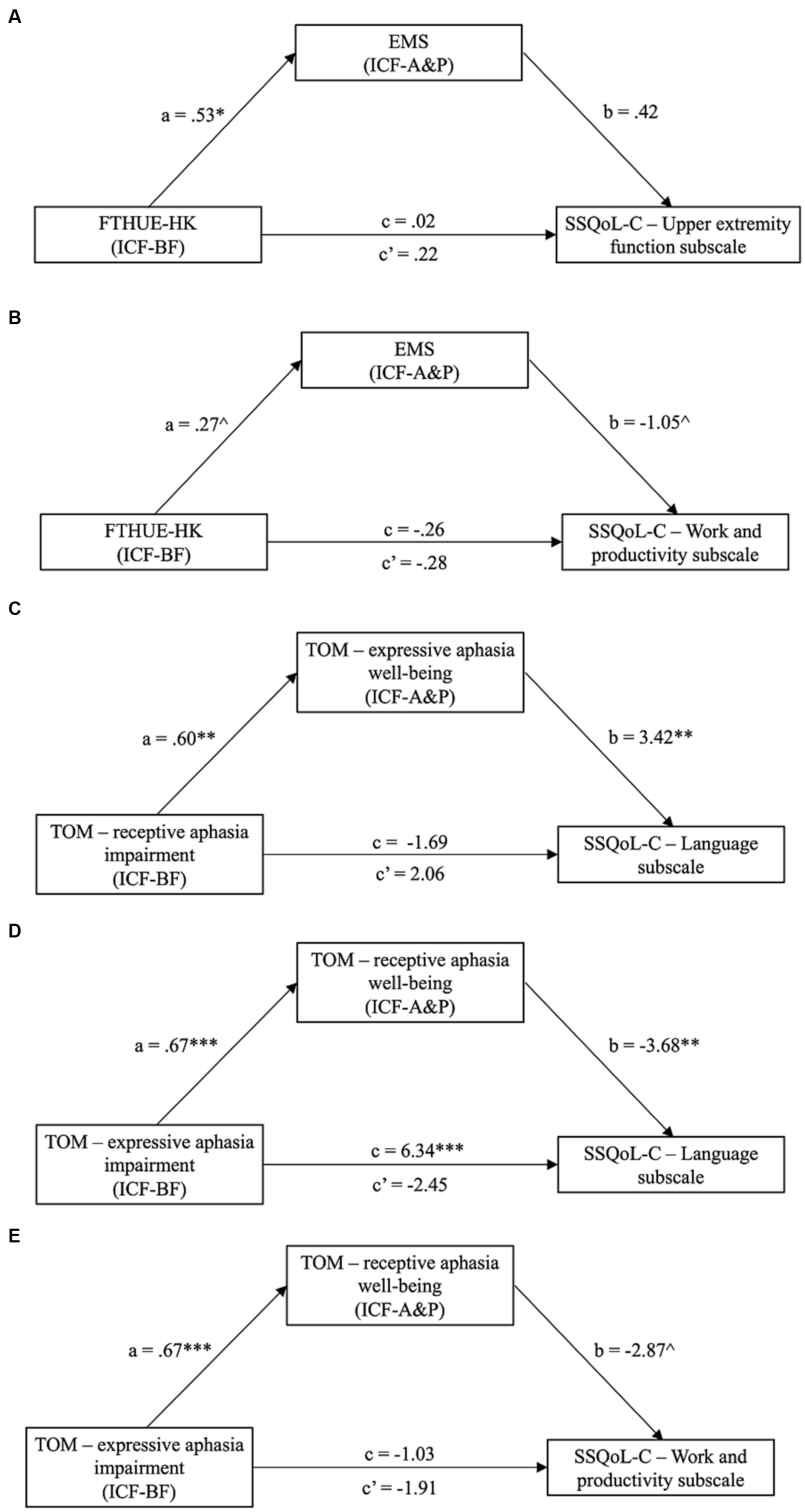
Significant models (Model **A** to **E** in the figure) from mediation analyses with ICF-A&P as the mediator and ICF-BF as the independent variable (IV); dependent variables (DV) are subscales from the SSQoL-C. Path a, b and c′ are indirect effects, while path c is the direct effect. ^*p* = 0.07; *p < 0.05; **p < 0.01; *** *p* < 0.001. CI, confidence interval; EMS, Elderly Mobility Scale; FTHUE-HK, Hong Kong version of the Functional Test for the Hemiplegic Upper Extremity; ICF-A&P, Activity and Participation; ICFBF, Body Function; SSQoL-C, Chinese version of the Stroke Specific Quality of Life Scale; TOM; Therapy Outcome Measures.

**Figure 4:**
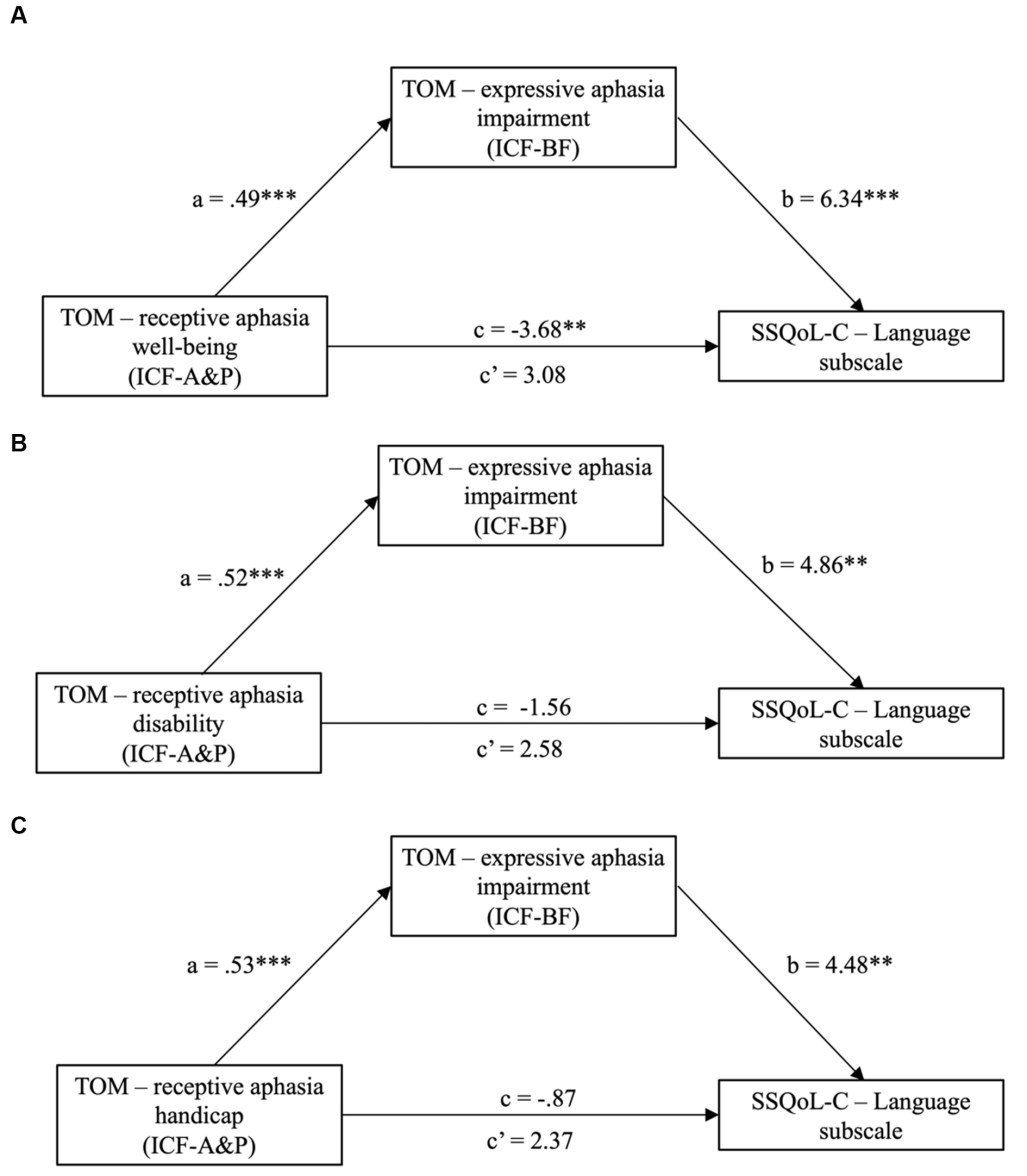
Significant models (Model **A** to **C** in the figure) from mediation analyses with ICF-BF as the mediator and ICF-A&P as the independent variable (IV); dependent variables (DV) are subscales from the SSQoL-C. Path a, b and c′ are indirect effects, while path c is the direct effect. **p < 0.01 and ***p < 0.001. CI, confidence interval; ICF-A&P, Activity and Participation; ICF-BF, Body Function; SSQoL-C, Chinese version of the Stroke Specific Quality of Life Scale; TOM; Therapy Outcome Measures.

All measures showing significant changes were extracted and entered to mediation analyses. Changes in the scores for the ICF-BF-and ICF-A&P-based measures and the secondary outcomes were grouped according to the model stipulated in Figure 2. Five mediation models with ICF-BF as the IV and ICF-A&P as the mediator were constructed to predict the secondary outcomes (Figure 3 and Supplementary Table S2). Secondary outcomes were satisfaction with quality of life (SSQoL) and goal attainment (GAS). The model with ICF-BF (i.e., TOM impairment scale for expressive aphasia score) as the IV, ICF-A&P (i.e., TOM well-being scale for receptive aphasia score) as the mediator, and SSQoL-C Language subscale as the DVs yielded the best prediction [*β* = −2.45, 95% confidence interval (CI) [−0.498, −0.305]]. Another model with ICF-BF (i.e., FTHUE-HK score) as the IV, ICF-A&P (i.e., EMS score) as the mediator, and the SSQoL-C Work and Productivity subscale score as the DV revealed significant results (*β* = −0.28, 95% CI [−0.758, −0.023]). Three models with the ICF-A&P score as the IV and the ICF-BF score as the mediator were constructed to predict the secondary outcomes (Figure 4 and Supplementary Table S3). The model with ICF-A&P (i.e., TOM well-being scale for receptive aphasia score) as the IV, ICF-BF (i.e., TOM impairment scale for expressive aphasia score) as the mediator, and the SSQoL-C Language subscale score as the DV yielded the best prediction (*β* = 3.08, 95% CI [0.637, 5.017]). In contrast, no significant models were found to be statistically significant when the patients’ goal attainment was used as the DV. Regardless of the DV, covariates such as age and sex were not significant factors in any of the significant models.

### 3.7. Qualitative analysis to triangulate SEM models

Content analyses were conducted on the sequence and number of intervention modules received by the patients (Table 3). In all three treatment phases, LE (beginning phase: 69.8%, middle phase: 76.9%, late phase: 82.2%) and speech (beginning phase: 42.3%, middle phase: 54.5%, late phase: 52.9%) intervention modules showed predominantly concurrent delivery of the ICF-BF and ICF-A&P components, i.e., BF|A&P. Different sequence patterns were identified for the UE intervention modules. There were increases in the delivery of ICF-BF modules from the beginning to the late phases (beginning phase: 40%, middle phase: 47.9%, late phase: 68.4%). UE treatments tended to focus on the concurrent delivery of intervention modules in the beginning phase, but their proportion decreased with time (beginning phase: 54%, middle phase: 43.8%, late phase: 26.3%) and with the increase in the proportion of patients undergoing ICF-BF treatments (beginning phase: 40%, middle phase: 47.9%, late phase: 68.4%).

**Table 3:** Content analyses on the types (BF or A&P) and sequences (BF➔A&P, A&P➔BF, and A&P|BF) of the intervention modules involving upper extremity, lower extremity, and speech received by the patients in the ICF-PARP in the beginning, middle, and late phases of the program.

Treatment contents in case note	Beginning phase, patterns included*:					Middle phase, patterns included*:					Late phase, patterns included*:				
	A	B	C	D	E	A	B	C	D	E	A	B	C	D	E
Upper extremity	54%	4%	0%	40%	2%	43.8%	6.3%	0%	47.9%	2.1%	26.3%	2.6%	2.6%	68.4%	0%
Lower extremity	69.8%	17%	3.8%	5.7%	3.8%	76.9%	3.8%	5.7%	3.8%	9.6%	82.2%	6.7%	2.2%	6.7%	2.2%
Speech	42.3%	7.7%	7.7%	19.2%	23.1%	54.5%	0%	0%	13.6%	31.8%	52.9%	5.9%	0%	11.8%	29.4%

The beginning phase is equivalent to 1st to 10th for a 30-session program or 1st to 16th for a 48-session program; the middle phase is equivalent to 11th to 20th or 17th to 32nd sessions; the late phase is equivalent to, i.e., 21st to 30th or 33rd to 48th sessions. BF, body function; A&P, activity and participation. Content and sequence patterns: (A) A&P|BF (integrative contents): BF and A&P contents, no A&P versus BF sequence is observed. (B) BF➔A&P (body function then activity and participation contents), BF contents occupy the prior 50% of the phase, A&P contents are added at the late 50% of the phase. (C) A&P➔BF (activity and participation then body function contents), A&P contents occupy the prior 50% of the phase, BF contents are added at the late 50% of the phase. (D) BF only. (E) A&P only.

## 4. Discussion

The current study aimed to investigate the effectiveness of an ICF-PSRP in enhancing patients’ ability to reintegrate into the community. Our results indicated improvements in almost all aspects of body functions (i.e., ICF-BF) and activity and participation (i.e., ICF-A&P), such as mobility and IADL, after implementation of the ICF-PSRP. The only exception was cognition, which did not show significant improvements. These improvements were comparable to those previously reported for various conventional post-stroke programs (40, 41) and ICF-based rehabilitation programs (11, 13). New findings from this study are that improvements in the ICF-BF and ICF-A&P scores, and their relationships, predicted patients’ satisfaction in different ways. The strongest prediction was found for interventions provided by speech therapists on expressive and receptive aphasia. Patients’ satisfaction with their quality of life related to language (SSQoL-C Language subscale) was predicted by improvements in the ERL function (ICF-BF, TOM Impairment Scale) and mediated by patients’ ERL improvements in daily life (ICF-A&P, TOM Well-Being Scale). The reciprocal relationships between ERL components, i.e., the ICF-A&P score as the predictor and the ICF-BF score as the mediator, also showed comparable predictability of patients’ satisfaction. The closed-looped predictor–mediator–outcome relationships in ERL may have been confounded by the overlapping measurement constructs among the instrument’s subscales. However, the patients’ satisfaction with their work-related quality of life gave a clear demonstration of the contributions of BF-A&P to the ICF model. UE function improvement (ICF-BF, FTHUE-HK), mediated by LE mobility improvement (ICF-A&P, EMS), was a significant predictor of the patients’ satisfaction with their work (SSQoL-C Work and Productivity subscale). Content analyses further supported a combined BF–A&P treatment approach throughout the program, and particularly during treatments targeting LE and speech, to support personalized treatments to achieve the patients’ goals that were set at the beginning of the program.

Patients showed significant improvements in various aspects after the ICF-based program. The results were consistent with those of previous post-stroke rehabilitation studies based on a one-group pre-and post-intervention design. Our results suggested that the goal-setting process and customized treatment content may have largely contributed to the positive outcomes. The goals set by the patients largely emphasised resuming life roles and community reintegration. Treatment contents and modules in the ICF-based program had been expanded to cater these diverse goals. For instance, outdoor walking training was offered to patients who targeted to walk better in their community, and simulated escalator training was offered to those who preferred to resume community living. Effective goal-setting and personalized treatment contents in post-stroke rehabilitation have been reported to result in enhanced motivation for behavioral changes, improved functional abilities, and the resumption of meaningful activities of daily life (42). The goals set by the patients determined the type, intensity, and duration of the interventions assigned by the case therapist. For instance, Patient A expressed a desire to return to his teacher role. Therefore, improving writing skills was identified as a core component to be addressed in this patient’s personalized treatment program. Patient A was assigned fine motor skill training, including ICF-BF – (e.g., hand grip and pinch grip strengthening) and ICF-A&P-related activities (e.g., fine motor exercises and writing tasks). The goals set and the subsequent personalized treatment arrangements were comparable to those described by Abarghuei, Mehraban (11). The goal set by the patient in the case study reported by Abarghuei, Mehraban (11) related to independent living in the community. Therefore, ICF-BF (e.g., splint and orthosis position) and ICF-A&P-related training (e.g., gait training) were assigned to meet his needs. In contrast to other studies, the ICF-based program we designed used a multidisciplinary approach to offer multidimensional treatments to patients. Other studies used single discipline such as physiotherapy or occupational therapy (12, 13). No statistically significant results were observed in the gender effects on the treatment outcomes, which were inconsistent with those revealed in previous studies. Studies indicated that females post-stroke patients were more prone to impact on their levels of ADL independence and mobility than their male counterpart (43). One study explained the gender effects could have been due to the older ages of female (for 4 years) than male patients at the first stroke episode (44), resulting in a slower and poorer function recovery. Another study suggested that the higher post-stroke depression rate among female than male patients could have attributed to their poorer functional recovery (45). The inconsistent findings might have been due to the small sample sizes of the male and female subgroups (i.e., 33 and 19, respectively), and no control of patients’ post-stroke depression.

Our findings that ICF-BF or ICF-A&P scores played mediating roles in determining the intervention outcomes are noteworthy. The ICF model does not stipulate specific relationship between these two components. In this study, we observed a tendency for the ICF-A&P score to play a mediating role. ICF-A&P score became significant mediator of the ICF-BF score when predicting patients’ satisfaction with their expressive and receptive language, work, and productivity. The findings are intuitive and consistent with those reported in non-ICF studies of post-stroke patients. First, in our ICF model, the improvement in UE function (ICF-BF) mediated by the improvement in LE function (ICF-A&P) predicted patients’ satisfaction with their work and productivity. Upper extremity and lower extremity functions are moderately correlated with the dynamic postural balance of post-stroke patients (46). Their dysfunctions have also been found to significantly hinder patients’ ability to return to work (47). In many job types, such as desktop service and computing, the ability to maneuver equipment with the upper extremities would be more challenging to regain than using a wheelchair to replace lower extremity mobility. Second, in another ICF model, expressive and receptive language improvements (ICF-BF) mediated by language improvements in daily life (ICF-A&P) predicted patients’ satisfaction with their language. Our results are consistent with those of previous studies, suggesting that the combination of ICF-BF and ICF-A&P in speech therapy allows patients to pursue social interactions and employment (48). Simulated life-related situations during therapy have been incorporated in the syntax and naming of training programs to enable post-stroke patients to resume their life roles (49).

However, the predictor and mediator roles of ICF-BF and ICF-A&P in satisfaction with expressive and receptive language were reversed. The analyses of the treatment program contents substantiated that the predictor–mediator roles would largely be influenced by the patients’ treatment goals set at the beginning of the program and, hence, the sequence of the treatment modules. Content analyses showed that most of the language-related treatment contents were a combination of the ICF-BF and ICF-A&P modules throughout the post-stroke program. The combined BF-A&P approach revealed in this study has its merit. On one hand, ICF-BF-related training is an essential treatment approach for enhancing the regaining of functions loss after a stroke (50). On the other hand, focusing on ICF-A&P has been found to promote the resumption of life roles after a stroke (49). More importantly, the combination of different types of intervention and breaking down the treatment goals can enhance patients’ motivation and their adherence to the treatment regime (51). Another study found that the breaking down of treatment goals and patients’ achievements and the provision of intermittent rewards empowered patients to experience their successes and internalize their treatment goals (52). We found that the upper extremity interventions tended to organize in patterns that began with a combined approach but ended with ICF-BF-related training. The main constraint observed for the program was that the low level of upper extremity function in patients impacted their engagement in ICF-A&P-related training. Another reason for the upper extremity interventions to show this trend was the relatively short length of the post-stroke program in this study, which did not cater to the extended recovery period required to regain upper extremity function (53).

There were several limitations of our study. First, the use of a non-randomized clinical trial and a small sample size (*N* = 52) may have biased the results pertaining to treatment effectiveness. Second, the non-significant results obtained for predicting goal attainment as a secondary treatment outcome were unexpected. Despite qualitative analyses indicating general increases in the pre-discharge ratings on the GAS, the instrument uses a 7-point Likert scale, which may have lowered its sensitivity to reflect the patients’ gains from the ICF-based program (54). A previous study concluded that changes in patients’ goal attainment levels could not be entirely captured with conventional clinical assessments (55). Alternative measures suggested to assess changes in goal attainment include the Canadian Occupational Performance Measure (56) and ICF-based goal statements with the ICF classification system codes (57). Third, the clinical instruments used to assess body function, activity, participation, and satisfaction with the language-related quality of life were non-ICF assessments. Some of these concepts used in clinical assessments, particularly those that were overgeneralized, may not have been described by the ICF model. For example, concepts such as “personal life” in the SSQoL could not be described using the ICF (58). These test items may have confounded the results and, hence, the interchanging predictor–mediator roles in the two ICF-models. Future studies using a randomized clinical trial format and the ICF Categorical Profile are recommended to further explore the effectiveness of the ICF-PSRP.

## 5. Conclusion

The current study explored the effect of the new ICF-PSRP in terms of enhancing patients’ community reintegration. The program was delivered by a multidisciplinary professional team. The results indicated that the goal-setting process and the combined treatment regime improved patients’ body function, activity, participation, and satisfaction with their quality of life. The treatment contents focused on upper extremity, lower extremity, and language functions. Significant treatment models explained that, in general, the patients’ improvements in their body functions, mediated by improvements in the activity of participation, predicted their levels of satisfaction with their quality of life in the community. The treatment goals set by the patients formed the basis for the multi-disciplinary team to select and organize the contents and flow of the intervention modules. The combination of training contents related to body function and activity and participation may be a common feature of future ICF-based rehabilitation programs.

## Data availability statement

The datasets presented in this article are not readily available because data may identify patients’ stroke and recovery conditions. Requests to access the datasets should be directed to ngai-kiu.wong@connect.polyu.hk.

## Ethics statement

The studies involving humans were approved by the Human Subjects Ethics Sub-committee of Hong Kong Polytechnic University. The studies were conducted in accordance with the local legislation and institutional requirements. The participants provided their written informed consent to participate in this study.

## Author contributions

MW, MC, Y-MN, and CC designed the study. MW performed data analysis and wrote the first draft of the manuscript. BL, SF, and CC supervised this study. MW, H-LY, and CC reviewed and edited the manuscript. All authors contributed to the article and approved the submitted version.
