# Supplementary material for: International Classification of Functioning, Disability, and Health-based rehabilitation program promotes activity and participation of post-stroke patients
Source: Front Neurol. 2023 Nov 3;14:1235500. doi: 10.3389/fneur.2023.1235500 (PMC10657202; doi:10.3389/fneur.2023.1235500)
Supplement: Supplementary file 1 [file Table_1.DOCX]

**Supplementary Materials**

*Table S1. Results of repeated measures analysis of variance (ANOVAs) on the scores of the Body Function (ICF-BF), Activity and Participation (ICF-A&P), and secondary outcome measures between the intake and the pre-discharge assessment occasions.*

| Measures | intake Scores (Mean, SD) | Pre-discharge Scores  (Mean, SD) | Repeated Measures ANOVAs |
| --- | --- | --- | --- |
| Body Function (ICF-BF) | | | |
| FTHUE-HK | 3.51 (2.30) | 3.98 (2.23) | *F*(1,51) = 18.15, *p* < .001***, η^2^_p_ = 0.26 |
| HK-OCS |  |  |  |
| Picture naming | 3.23 (1.52) | 3.21 (1.48) | *F*(1,51) = 0.06, *p* = .811, η^2^_p_ = 0 |
| Semantics | 2.65 (0.88) | 2.69 (0.88) | *F*(1,51) = 0.31, *p* = .580, η^2^_p_ = 0.01 |
| Orientation | 3.67 (0.96) | 3.73 (1.17) | *F*(1,51) = 0.21, *p* = .652, η^2^_p_ = 0 |
| Visual field-left | 1.58 (0.80) | 1.54 (0.83) | *F*(1,51) = 0.28, *p* = .598, η^2^_p_ = 0.01 |
| Visual field-right | 1.69 (0.70) | 1.69 (0.73) | *F*(1,51) = 0.01, *p* = .940, η^2^_p_ = 0 |
| Sentence reading | 17.35 (7.78) | 17.85 (7.45) | *F*(1,51) = 0.60, *p* = .443, η^2^_p_ = 0.01 |
| Number writing | 2.30 (1.13) | 2.33 (1.18) | *F*(1,51) = 0.08, *p* = .773, η^2^_p_ = 0 |
| Calculation | 3.36 (0.99) | 3.31 (0.99) | *F*(1,51) = 0.06, *p* = .801, η^2^_p_ = 0 |
| Attention | 36.33 (18.56) | 39.13 (16.33) | *F*(1,51) = 6.14, *p* = .017*, η^2^_p_ = 0.11 |
| Praxis | 9.67 (3.77) | 9.94 (3.78) | *F*(1,51) = 0.74, *p* = .394, η^2^_p_ = 0.01 |
| Verbal memory | 3.13 (1.34) | 3.40 (1.36) | *F*(1,51) = 2.23, *p* = .142, η^2^_p_ = 0.04 |
| Episodic memory | 3.41 (1.17) | 3.45 (1.22) | *F*(1,50) = 0.28, *p* = .598, η^2^_p_ = 0.01 |
| Executive test | 1.86 (4.30) | 1.84 (4.65) | *F*(1,50) = 0, *p* = .978, η^2^_p_ = 0 |
| MMT-LE | 6.43 (3.38) | 6.98 (3.27) | *F*(1,51) = 11.45, *p* = .001**, η^2^_p_ = 0.18 |
| TOM |  |  |  |
| Receptive aphasia – impairment | 3.90 (1.69) | 4.09 (1.49) | *F*(1,34) = 4.61, *p* = .017*, η^2^_p_ = 0.16 |
| Expressive aphasia – impairment | 3.59 (1.74) | 3.94 (1.44) | *F*(1,34) = 19.50, *p* < .001***, η^2^_p_ = 0.36 |
| Dysarthria – impairment | 4.23 (0.76) | 4.60 (0.58) | *F*(1,29) = 15.67, *p* < .001***, η^2^_p_ = 0.35 |
| Activity and Participation (ICF-A&P) | | | |
| EMS | 12.50 (3.88) | 14.87 (3.33) | *F*(1,51) = 89.61, *p* < .001***, η^2^_p_ = 0.64 |
| iADL-CV | 8.33 (6.53) | 11.40 (7.25) | *F*(1,51) = 41.87, *p* < .001***, η^2^_p_ = 0.45 |
| mBI-C | 74.21 (20.06) | 83.85 (16.98) | *F*(1,51) = 81.72, *p* < .001***, η^2^_p_ = 0.62 |
| TOM |  |  |  |
| Receptive aphasia – disability | 3.96 (1.60) | 4.33 (1.19) | *F*(1,34) = 17.90, *p* < .001***, η^2^_p_ = 0.35 |
| Receptive aphasia – handicap | 3.97 (1.57) | 4.37 (1.11) | *F*(1,34) = 19.23, *p* < .001***, η^2^_p_ = 0.36 |
| Receptive aphasia – well-being | 3.91 (1.69) | 4.20 (1.37) | *F*(1,34) = 9.13, *p* = .005**, η^2^_p_ = 0.16 |
| Expressive aphasia – disability | 3.66 (1.66) | 4.20 (1.23) | *F*(1,34) = 26.60, *p* < .001***, η^2^_p_ = 0.44 |
| Expressive aphasia – handicap | 3.69 (1.61) | 4.23 (1.16) | *F*(1,34) = 28.78, *p* < .001***, η^2^_p_ = 0.46 |
| Expressive aphasia – well-being | 3.63 (1.73) | 4.10 (1.34) | *F*(1,34) = 21.21, *p* < .001***, η^2^_p_ = 0.38 |
| Dysarthria – disability | 4.33 (0.65) | 4.87 (0.32) | *F*(1,29) = 27.60, *p* < .001***, η^2^_p_ = 0.49 |
| Dysarthria – handicap | 4.35 (0.63) | 4.85 (0.33) | *F*(1,29) = 27.19, *p* < .001***, η^2^_p_ = 0.48 |
| Dysarthria – well-being | 4.23 (0.77) | 4.68 (0.53) | *F*(1,29) = 15.77, *p* < .001***, η^2^_p_ = 0.35 |
| Secondary Outcome Measures | | | |
| GAS | -2 (same for all patients) | 0.50 (1.30) | *F*(1,44) = 167.23, *p* < .001***, η^2^_p_ = 0.79 |
| SSQoL-C |  |  |  |
| Family role | 7.42 (1.99) | 8.16 (2.28) | *F*(1,51) = 4.15, *p* = .047*, η^2^_p_ = 0.08 |
| Language | 19.88 (5.33) | 21.42 (4.75) | *F*(1,51) = 12.34, *p* = .001**, η^2^_p_ = 0.20 |
| Mobility | 21.73 (5.48) | 23.72 (4.50) | *F*(1,51) = 9.26, *p* = .004**, η^2^_p_ = 0.15 |
| Self-care | 18.37 (4.95) | 19.42 (4.06) | *F*(1,51) = 2.30, *p* = .135, η^2^_p_ = 0.04 |
| Social role | 12.98 (3.50) | 14.69 (3.86) | *F*(1,51) = 11.42, *p* = .001**, η^2^_p_ = 0.18 |
| Upper Extremity function | 18.48 (4.20) | 19.63 (3.85) | *F*(1,51) = 3.85, *p* = .055^, η^2^_p_ = 0.07 |
| Work and productivity | 8.47 (2.94) | 9.78 (2.12) | *F*(1,51) = 4.61, *p* = .037*, η^2^_p_ = 0.08 |

Note: ^*p* = 0.07; **p* < .05; ***p* < .01; **** p* < .001. Effect sizes using partial eta squared (η^2^_p_) were reported for the ANOVA results. Small, medium and large effect sizes of the results were represented by 0.01, 0.06, and 0.14 respectively. EMS, Elderly Mobility Scale; FTHUE-HK, Hong Kong version of the Functional Test for the Hemiplegic Upper Extremity; GAS, Goal Attainment Scale; HK-OCS, The Hong Kong version of the Oxford Cognitive Screen; iADL-CV, Chinese Version of the Lawton Instrumental Activities of Daily Living ; mBI-C, Chinese version of Modified Barthel Index; MMT-LE, Manual Muscle Testing – Lower Extremity; SSQoL-C, Chinese version of the Stroke Specific Quality of Life Scale; TOM; Therapy Outcome Measures.

*Table S2. Results of mediation analyses with BF factors as independent variables, secondary outcomes as dependent variables, and A&P factors as mediators.*

| Independent variable (X) | Dependent variable (Y) | Mediator (M) | Indirect effects | | | | | | | | | | Direct effect | | | |
| --- | --- | --- | --- | --- | --- | --- | --- | --- | --- | --- | --- | --- | --- | --- | --- | --- |
|  |  |  | Path a (X to M) | | | | Path b (M to Y) | | | | Path c’ (X to Y) | | Path c | | | |
|  |  |  | β | SE | *t* | *p* | β | SE | *t* | *p* | β | 95% CI [lower, upper] | β | SE | *t* | *p* |
| FTHUE-HK | SSQoL-C – Upper extremity function | EMS | .53 | .20 | 2.66 | .010 | .42 | .35 | 1.20 | .237 | .22 | [.004, .627] | .02 | .52 | .033 | .974 |
| TOM – receptive aphasia impairment | SSQoL-C – Language | TOM – expressive aphasia well-being | .60 | .22 | 2.78 | .009 | 3.42 | 1.04 | 3.30 | .002 | 2.06 | [.179, 4.466] | -1.69 | 1.43 | -1.18 | .247 |
| TOM – expressive aphasia impairment |  | TOM – receptive aphasia well-being | .67 | .17 | 3.97 | .000 | -3.68 | 1.05 | -3.52 | .001 | -2.45 | [-4.498, -.305] | 6.34 | 1.22 | 5.18 | .000 |
| FTHUE-HK | SSQoL-C – Work and productivity | EMS | .27 | .14 | 1.93 | .060 | -1.05 | .53 | -1.99 | .053 | -.28 | [-.758, -.023] | -.26 | .53 | -.50 | .619 |
| TOM – expressive aphasia impairment |  | TOM – receptive aphasia well-being | .67 | .17 | 3.97 | .000 | -2.87 | 1.42 | -2.02 | .052 | -1.91 | [-3.674, -.134] | -1.03 | 1.67 | -.62 | .539 |

Note: Path a (i.e. from ICF-BF factors to ICF-A&P factors), b (i.e. from ICF-A&P factors to secondary outcomes) and c (i.e. from ICF-BF factors to secondary outcomes with the presence of mediator) were indirect effects, while Path c’ (i.e. from BF factors to secondary outcomes without the presence of mediator) was the direct effect. CI, confidence interval; EMS, Elderly Mobility Scale; FTHUE-HK, Hong Kong version of the Functional Test for the Hemiplegic Upper Extremity; SSQoL-C, Chinese version of the Stroke Specific Quality of Life Scale; TOM; Therapy Outcome Measures.

*Table S3. Results of mediation analyses with A&P factors as independent variables, secondary outcomes as dependent variables, and BF factors as mediators.*

| Independent variable (X) | Dependent variable (Y) | Mediator (M) | Indirect effects | | | | | | | | | | Direct effect | | | |
| --- | --- | --- | --- | --- | --- | --- | --- | --- | --- | --- | --- | --- | --- | --- | --- | --- |
|  |  |  | Path a (X to M) | | | | Path b (M to Y) | | | | Path c’ (X to Y) | | Path c | | | |
|  |  |  | β | SE | *t* | *p* | β | SE | *t* | *p* | β | 95% CI [lower, upper] | β | SE | *t* | *p* |
| TOM – receptive aphasia well-being | SSQoL-C – Language | TOM – expressive aphasia impairment | .49 | .12 | 3.97 | .000 | 6.34 | 1.22 | 5.18 | .000 | 3.08 | [.637, 5.017] | -3.68 | 1.05 | -3.52 | .001 |
| TOM – receptive aphasia disability |  |  | .52 | .13 | 4.04 | .000 | 4.86 | 1.42 | 3.43 | .002 | 2.58 | [1.101, 5.442] | -1.56 | 1.31 | -1.19 | .241 |
| TOM – receptive aphasia handicap |  |  | .53 | .12 | 4.29 | .000 | 4.48 | 1.47 | 3.05 | .005 | 2.37 | [.964, 5.177] | -.87 | 1.30 | -.67 | .508 |

Note: Path a (i.e. from ICF-A&P factors to ICF-BF factors), b (i.e. from ICF-BF factors to secondary outcomes) and c (i.e. from A ICF-&P factors to secondary outcomes with the presence of mediator) were indirect effects, while Path c’ (i.e. from ICF-A&P factors to secondary outcomes without the presence of mediator) was the direct effect. CI, confidence interval; EMS, Elderly Mobility Scale; FTHUE-HK, Hong Kong version of the Functional Test for the Hemiplegic Upper Extremity; SSQoL-C, Chinese version of the Stroke Specific Quality of Life Scale; TOM; Therapy Outcome Measures.
